# Computed terahertz near-field mapping of molecular resonances of lactose stereo-isomer impurities with sub-attomole sensitivity

**DOI:** 10.1038/s41598-019-53366-0

**Published:** 2019-11-15

**Authors:** Kiwon Moon, Youngwoong Do, Hongkyu Park, Jeonghoi Kim, Hyuna Kang, Gyuseok Lee, Jin-Ha Lim, Jin-Woo Kim, Haewook Han

**Affiliations:** 1 Department of Electrical Engineering, Pohang University of Science and Technology, Pohang, 37673 Republic of Korea; 2 Department of Biological and Agricultural Engineering and Institute for Nanoscience and Engineering, University of Arkansas, Fayetteville, Arkansas 72701 USA; 3 Present Address: THz Basic Research Section, Electronics and Telecommunications Research Institute, Daejeon, 34129 Republic of Korea; 4 Present Address: SK Hynix Inc., Icheon, 17336 Republic of Korea; 5 Present Address: Samsung Electronics, Suwon, 16677 Republic of Korea; 6 Present Address: Korea Brain Research Institute, Daegu, 41062 Republic of Korea

**Keywords:** Terahertz optics, Scanning probe microscopy

## Abstract

Terahertz near-field microscopy (THz-NFM) could locally probe low-energy molecular vibration dynamics below diffraction limits, showing promise to decipher intermolecular interactions of biomolecules and quantum matters with unique THz vibrational fingerprints. However, its realization has been impeded by low spatial and spectral resolutions and lack of theoretical models to quantitatively analyze near-field imaging. Here, we show that THz scattering-type scanning near-field optical microscopy (THz s-SNOM) with a theoretical model can quantitatively measure and image such low-energy molecular interactions, permitting computed spectroscopic near-field mapping of THz molecular resonance spectra. Using crystalline-lactose stereo-isomer (anomer) mixtures (*i*.*e*., α-lactose (≥95%, w/w) and β-lactose (≤4%, w/w)), THz s-SNOM resolved local intermolecular vibrations of both anomers with enhanced spatial and spectral resolutions, yielding strong resonances to decipher conformational fingerprint of the trace β-anomer impurity. Its estimated sensitivity was ~0.147 attomoles in ~8 × 10^−4^ μm^3^ interaction volume. Our THz s-SNOM platform offers a new path for ultrasensitive molecular fingerprinting of complex mixtures of biomolecules or organic crystals with markedly enhanced spatio-spectral resolutions. This could open up significant possibilities of THz technology in many fields, including biology, chemistry and condensed matter physics as well as semiconductor industries where accurate quantitative mappings of trace isomer impurities are critical but still challenging.

## Introduction

Terahertz time-domain spectroscopy (THz-TDS) has offered a powerful tool to probe low-energy intermolecular interactions of biomolecules, and could coherently measure macroscopic dielectric functions for material-specific spectroscopic information by detecting the phase as well as the amplitude of sub-picosecond terahertz (THz) pulses^1^. However, THz-TDS inevitably measures an ensemble average of resonances from microscopic sub-domains due to the diffraction limit. THz near-field microscopy (THz-NFM)^2–10^ and THz scanning tunneling microscopy (THz-STM)^11–13^ have emerged to improve resolutions below diffraction limits^14–23^. Particularly, THz scattering-type scanning near-field optical microscopy (THz s-SNOM), which combines atomic force microscope (AFM) and THz-TDS, showed promise for quantitative spectroscopic imaging and sensing with enhanced spatial resolutions to probe low-energy molecular vibration dynamics below diffraction limits^3,5,8–10^. However, the quantitative sensing and imaging of molecular resonance is still difficult to attain because of a trade-off between sensitivity and spatial resolution as well as limited theoretical models for the quantitative analysis of THz near-field imaging^8,24–26^. Analytical models for the mid- and near-infrared s-SNOM have been reported, including semi-analytical models using empirical fitting parameters^27,28^; however, no such models have been demonstrated for quantitative s-SNOM due to the challenge to decipher local THz permittivity from near-field measurements.

Here, we present a new platform for a quantitative coherent broadband THz pulse spectroscopic mapping of molecular conformational dynamics of biomolecules. This is realized by uniquely combining a THz s-SNOM capable of near-field imaging with substantially enhanced resolutions and a rigorous theoretical model based on the line dipole image method (LDIM) with quasi-electrostatic boundary conditions^8,9^ to compute the local permittivity mapping of THz molecular resonance spectra. Assessing its capability with a mixture of two crystalline stereo-isomers (*i*.*e*., α- and β-anomers) of a common carbohydrate, lactose, we demonstrate a computed mapping of broadband complex dielectric function by resolving local intermolecular vibrations of each anomer at unprecedented sensitivity and spatio-spectral resolutions, enabling highly sensitive, broadband molecular fingerprinting of biomolecules or organic crystals in their complex mixtures. By demonstrating highly sensitive quantitative mappings of a small amount of isomer impurities (*i*.*e*., β-lactose) in the anomer mixture, we also show the promise of our THz s-SNOM technical platform for trace analysis of impurities in organic crystals, particularly isomer and polymorphic impurities in organic semiconducting materials, which critically impact the performance of organic thin-film transistors^29^.

## Results and discussion

### THz s-SNOM platform for biomolecular fingerprinting

**Figure 1:**
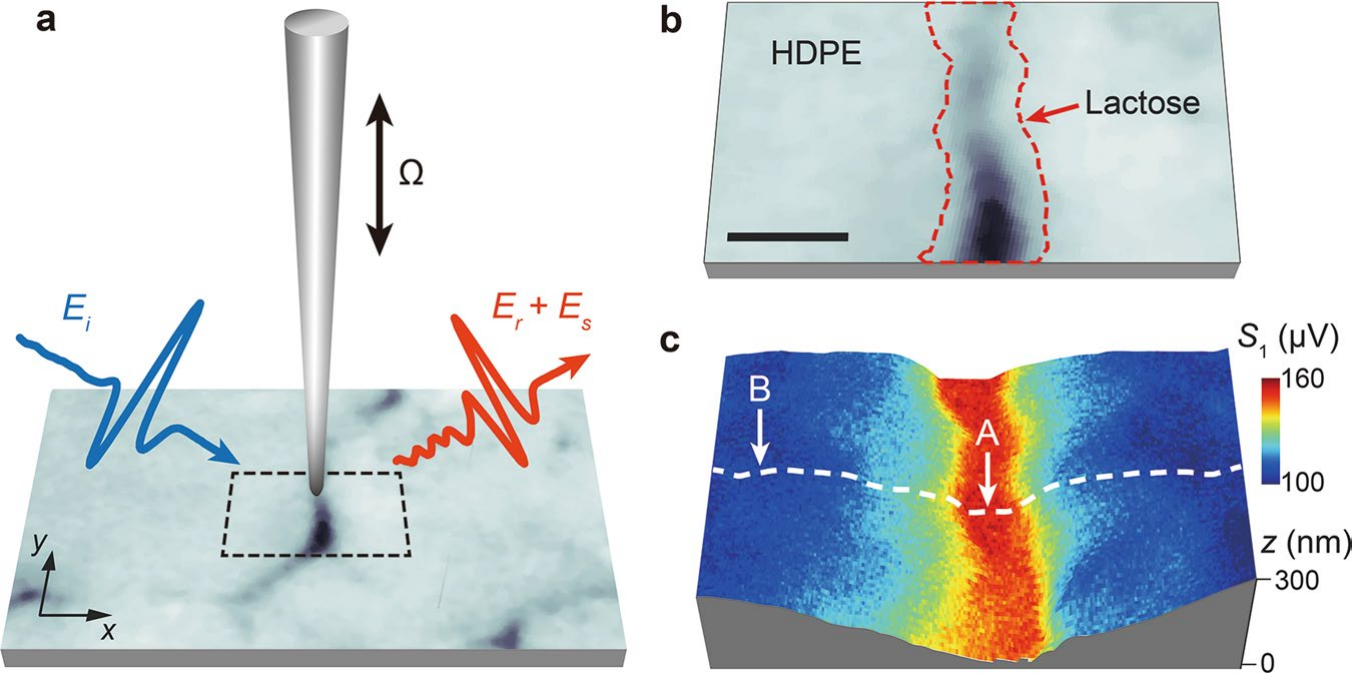
THz s-SNOM system for highly sensitive, quantitative sensing and imaging of molecular fingerprints of biomolecules. (**a**) Schematic showing our experimental set-up. A homemade tungsten probe on the sample surface simultaneously works for tapping-mode AFM and THz NFM. The probe length is >4 mm and its tip diameter ~600 nm. The probe tip-sample distance is precisely controlled by AFM (see Methods). Dashed black box indicates the field of interest in the sample pellet, in which crystalline lactose is mixed with high-density polyethylene (HDPE) (see Methods). The crystalline lactose is in the mixture of two stereo-isomers, *i*.*e*., α-lactose (≥95%, w/w) and β-lactose (≤4%, w/w) anomers. *E*_i_, incident field; *E*_s_, scattered field; *E*_r_, specula_r_ly reflected field; Ω, dithering frequency of the AFM probe. (**b**) Optical microscopic image of the sample area (dashed black box in a) where crystalline lactose anomers (dashed red area) are embedded between HDPE. The scale bar, 3 μm. (**c**) Sample topography (*z*) and integrated THz spectral amplitude (*S*_1_) of the first-harmonic scattering signal. Near-field measurements are conducted along the dashed white line as well as two points (A and B) on the dashed line.

A schematic of our THz s-SNOM system, which simultaneously implements AFM topographic scanning and THz near-field imaging of the sample, is depicted in Fig. 1a. While a homemade tapping-mode AFM raster-scans sample surface, THz near-field pulse scattered by a metallic AFM probe (*i*.*e*., a tungsten probe) is measured in the far-field region. A probe with a relatively large apex size (*i*.*e*., an apex diameter of **~**600 nm) is selected in this study in order to enhance scattering efficiencies on samples with low refractive indices (*e*.*g*., lactose). The probe-sample distance is precisely controlled by AFM and the photocurrent output of THz detector is demodulated by the first harmonic of the probe-tapping frequency with a lock-in amplifier. The first harmonic demodulation is used in this study to obtain more accurate molecular resonance frequencies due to its highest signal-to-noise ratio compared to higher harmonics^30^. By scanning the delay line of THz-TDS, the scattered pulse is measured in the time domain for near-field spectroscopy. In our experiments, crystalline lactose sample in the mixture of α-lactose (≥95%, w/w) and β-lactose (≤4%, w/w) anomers is mixed with high-density polyethylene (HDPE) to make its pellet for THz s-SNOM measurements. HDPE is chosen because it has no resonance peaks in the frequency range (0.1–2.5 THz) of our THz-TDS system. The pellet thickness is controlled to be ~3 mm to separate reflections from the sample backside (see Methods for further details of microscope setup and sample preparation).

Figure 1b shows the optical image of the field of interest in the lactose-HDPE sample (square in the dashed black line in Fig. 1a), where lactose is embedded between HDPE (the area in dashed red line). Figure 1c shows the integrated image of AFM topography (*z*) and THz near-field peak intensity (*S*_1_) mapping within the sample field. The high-intensity region in the middle of the image corresponds to the area with lactose in Fig. 1b. The permittivity of lactose is higher than that of HDPE, confirming the existence of lactose in the high near-field intensity area.

### THz s-SNOM biomolecular sensing and fingerprinting capabilities

**Figure 2:**
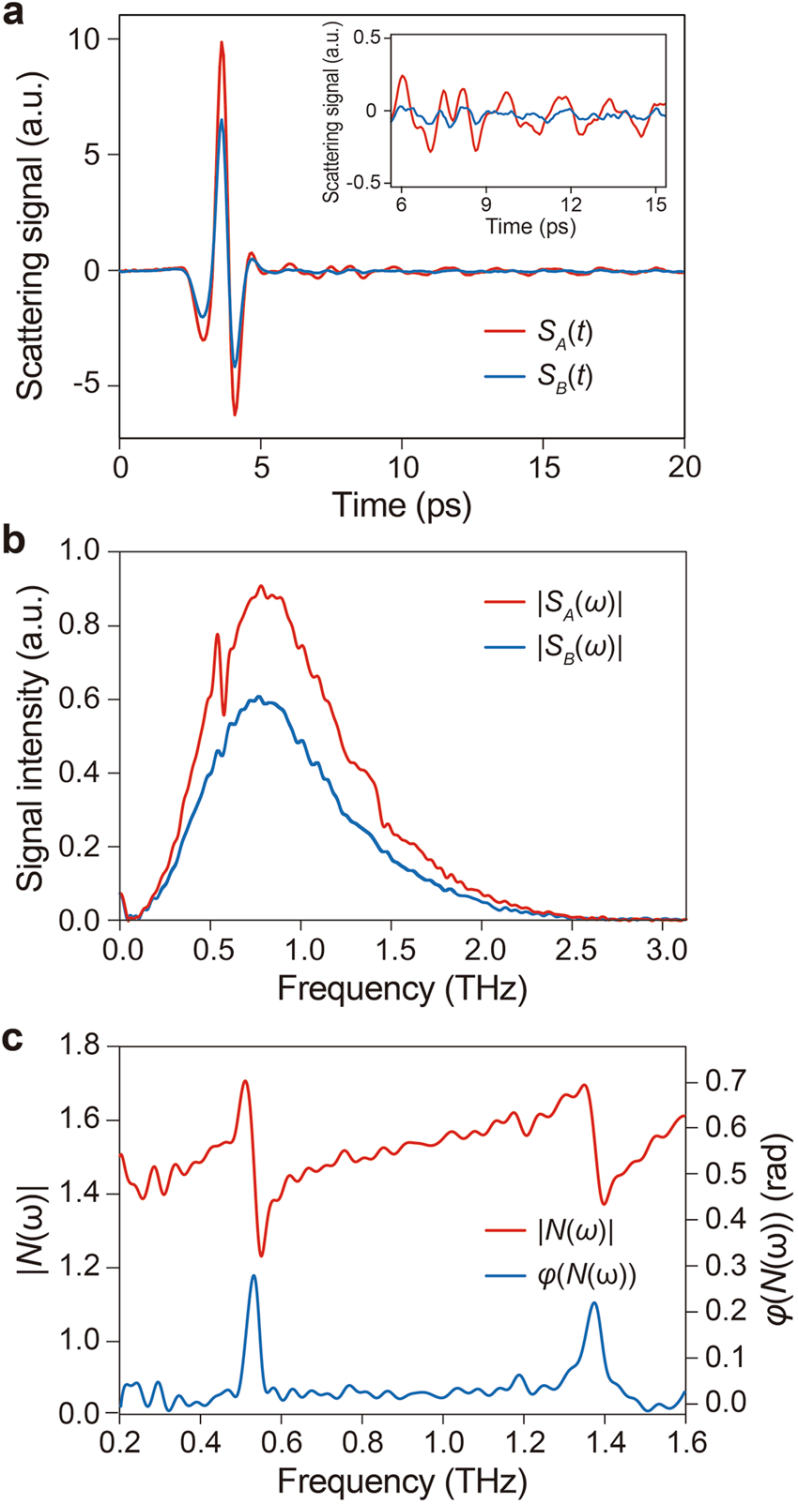
THz molecular resonance spectra of lactose anomer mixtures by THz s-SNOM. (**a**) Time-domain scattering signal at the point A (lactose, *S*_A_(*t*), red line) and B (HDPE, *S*_B_(*t*), blue line) in Fig. 1c. Inset shows the magnification of the transient oscillations after the main peak. (**b**) Fast Fourier Transform (FFT) amplitude of the scattering signal at the point A (lactose, *S*_A_(*ω*), red line) and B (HDPE, *S*_B_(*ω*), blue line) in Fig. 1c. (**c**) The amplitude (|*N*(*ω*)|, red line) and phase (*φ*(*N*(*ω*)), blue line) of the normalized spectrum (*N*(*ω*)) that is defined to be *N*(ω) = *S*_A_(*ω*)/*S*_B_(*ω*).

Time-domain scattering pulses (*S*(*t*)) and their fast Fourier transform (FFT) amplitude spectra (*S*(*ω*)) on the points A (lactose) and B (HDPE) in Fig. 1c are shown in Fig. 2a,b, respectively. Estimating the normalized spectrum (*N*(*ω*) = *S*_A_(*ω*)/*S*_B_(*ω*)), at least two characteristic resonance peaks can be clearly identified from lactose (Fig. 2c), which are not present in HDPE. The estimated *N*(ω) is, in turn, used to compute complex permittivity of lactose by applying the LDIM^8,9^ (see Methods and Supplementary Information for details of the LDIM).

**Figure 3:**
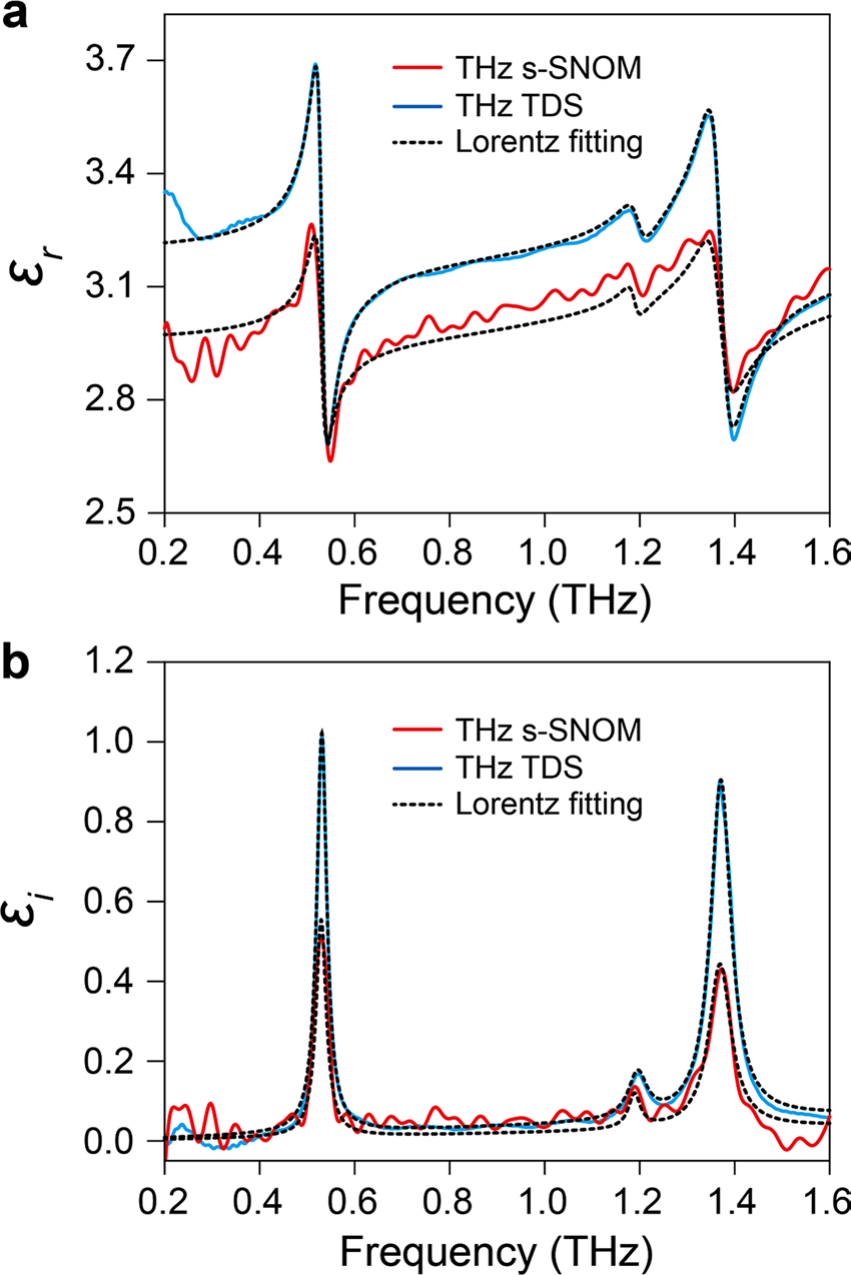
Computed local permittivity of lactose anomer mixtures: THz s-SNOM vs THz-TDS. (**a**,**b**) Real local permittivity (**a**, *ε*_r_) and imaginary local permittivity (**b**, *ε*_i_) extracted from THz s-SNOM near-field measurement (red lines) and reference THz-TDS measurement of the pure lactose pellet (blue lines). The *N*(*ω*) from the near-field measurement at the point A in Fig. 1c is used to compute the complex local permittivity of lactose anomer mixtures by applying the line dipole image method (LDIM) (see Methods) and compared with that of the reference THz-TDS measurement with a pure lactose pellet. Dotted black lines show the Lorentz fitting of the experimental results.

The complex local permittivity of lactose is computed from the near-field measurement by THz s-SNOM (at the point A in Fig. 1c) and compared with the permittivity of a reference THz-TDS measurement with a pure lactose pellet (see Methods) to evaluate THz s-SNOM sensing capabilities (Fig. 3a,b). The computed local permittivity from the near-field measurement (red lines) shows that its trends of amplitudes and phases are in good agreement with the permittivity from the reference TDS measurement (blue lines). Fitted to the triple Lorentz dispersion model (dashed black lines), both yield three distinct molecular resonances near 0.531 THz, 1.188 THz, and 1.371 THz (see Supplementary Information for details). These are in accordance with previously reported characteristic resonances of lactose anomers, *i*.*e*., the resonance at 0.531 THz due to an external hindered rotational mode along the B-axis of α-lactose crystal with intermolecular hydrogen-bond networks^31,32^, that at 1.371 THz due to inter- and intra-molecular vibrations in α-lactose^32^, and that at 1.188 THz due to β-lactose^33^. These results demonstrate the capability of THz s-SNOM not only to accurately measure local biomolecular resonances but also to detect them with high sensitivity considering that β-lactose anomer impurity consists of only ≤4% of the lactose sample.

**Figure 4:**
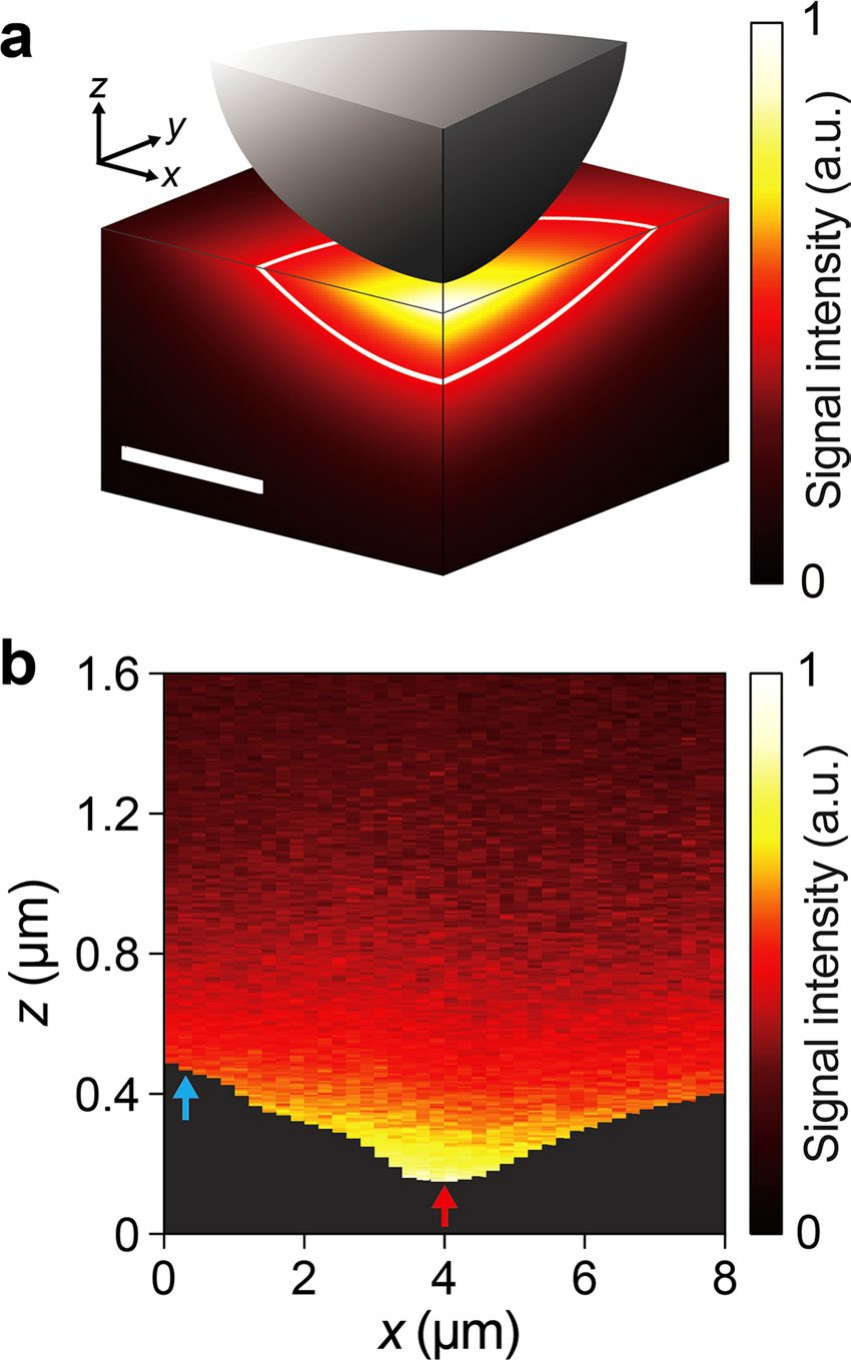
Sensitivity and biomolecular fingerprinting capability of THz s-SNOM. (**a**) Near-field distribution around the AFM probe tip. The dipole moment distribution obtained by LDIM is used to calculate the near-field distribution with the complex permittivity at the resonance frequency (1.188 THz) of β-lactose in Fig. 3. The sphere approximates the near-field probe. Solid white line shows the boundary of the field intensity penetration under 1/*e* cutoff criterion. The penetration volume is considered as the probe-sample interaction volume. The scale bar, 100 nm. (**b**) Experimentally measured near-field distribution on the lactose-HDPE sample surface (along the dotted white line in Fig. 1c) as a function of probe position (*x*) and probe-sample distance (*z*). Red arrow at *x* = 4 μm and blue arrow at *x* = 0.2 μm correspond to the point A (lactose anomer mixtures) and the point B (HDPE) in Fig. 1c, respectively.

THz s-SNOM’s sensing capability is further assessed by estimating THz near-field distribution around the probe apex (Fig. 4a). The quasi-electrostatic image theory was used to calculate the near-field distribution from the dipole moment distribution obtained by LDIM. Based on the near-field intensity distribution, the depth of probe-sample interaction is 40 nm (*z*-axis) and its widths are 235 nm (*x*-axis) and 371 nm (*y*-axis) under the 1/*e* cutoff criterion. This indicates that the THz s-SNOM system can measure local conformational dynamics of ~3.328 attomoles of α-lactose in ~8 × 10^−4^ μm^3^ interaction volume (see Supplementary Information for details of estimating the interaction volume and the detection sensitivity), assuming lactose powder is fully saturated in the volume and the probe-sample interaction volume is same as the THz near-field penetration volume. Moreover, the clear identification of molecular fingerprint of trace β-lactose anomer impurity (Fig. 3) implies that its sensitivity can be ~0.147 attomoles (see Supplementary Information for details of the detection sensitivity estimation), demonstrating unprecedentedly high sensitivity of THz s-SNOM. The true detection sensitivity could be even lower considering that it is implausible to fully saturate any powder mixture in its pellet and that the true volume of crystalline lactose should be less than the packing volume of the lactose powder in the pellet (see Methods for details of sample pellet preparation). Of note is that such sensitive sensing is possible even in the mixture of two lactose anomers, implying the promise of THz s-SNOM for highly sensitive, broadband molecular fingerprinting of biomolecules in their complex mixtures. This indicates great potential of THz s-SNOM as an alternative analytical tool for qualitative and quantitative trace analyses of organic crystals, particularly to detect a small amount of their isomer and polymorphic impurities that are still very challenging to analyze by conventional spectroscopic tools. Such residual impurities critically influence the properties and functions of pharmaceuticals as well as organic semiconducting crystals. For example, isomer impurities in organic semiconductors significantly impact the performance of organic thin-film transistors by promoting the formation of electronic gap states and ultimately reducing electrical conductivity^29^. Figure 4b shows the scattered field as a function of probe position (*x*) and probe-sample distance (*z*). The material contrast is significant when the probe is near the surface, confirming near-field interactions that enable deciphering local conformational dynamics of lactose.

### Computed THz near-field mapping of biomolecules

**Figure 5:**
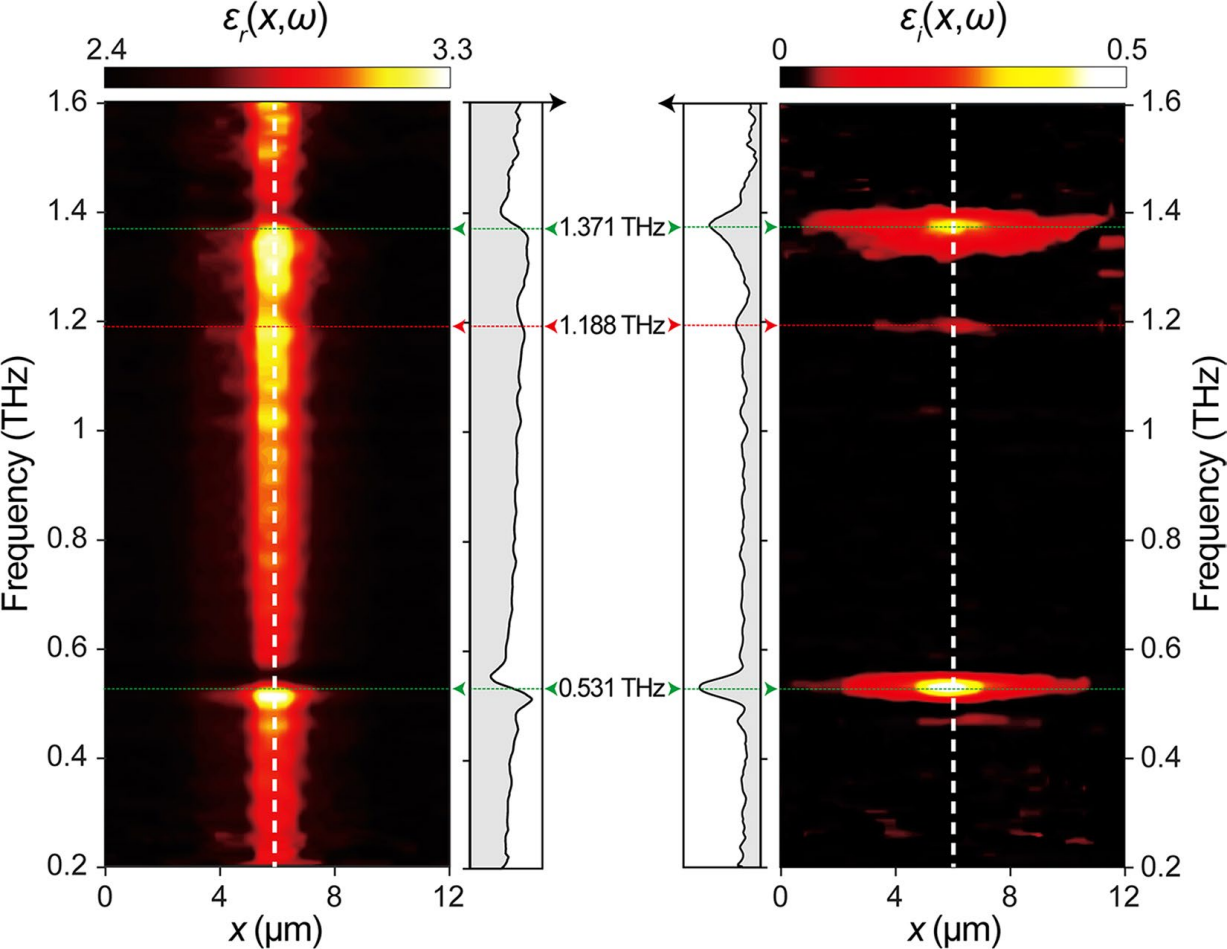
Computed spectroscopic near-field mapping of THz molecular resonance spectra of lactose anomer mixtures by THz s-SNOM. Spatio-spectral mappings of real permittivity (left, *ε*_r_(*x*,*ω*)) and imaginary permittivity (right, *ε*_i_(*x*,*ω*)) based upon the complex permittivity across the lactose sample. Dashed white lines correspond to the dashed white line in the lactose region in Fig. 1c. Dashed green lines and green arrows indicate resonances at 0.531 THz (bottom) and 1.371 THz (top) of α-lactose. Dashed red line and red arrow indicate a resonance at 1.188 THz of β-lactose.

Spatio-spectral mappings of the real (Fig. 5, left, *ε*_r_(*x*,*ω*)) and imaginary (Fig. 5, right, *ε*_i_(*x*,*ω*)) parts of complex permittivity are obtained based upon complex permittivity extracted from scattering signals across the lactose sample (*i*.*e*., along the dashed white line in Fig. 1c). Not only two strong resonances from α-lactose at 0.531 THz and 1.371 THz (dashed green lines and arrows in Fig. 5) but also a weak resonance from β-lactose anomer at 1.188 THz (dashed red line and red arrow in Fig. 5) are clearly revealed both in real and imaginary mappings. The apparent spatial resolution is ~600 nm (*λ*/500) at 1 THz and is frequency-independent. Local molecular vibrations of two lactose anomers in their mixture can thus be imaged and fingerprinted at markedly enhanced resolutions by our THz s-SNOM system.

## Conclusion

In summary, the development of a THz s-SNOM platform, capable of quantitative sensing and imaging of low-energy vibrational dynamics of biomolecules, opens the door to a new experimental domain in nano/biosciences. Now, molecular fingerprints of biomolecules in their complex mixtures can be probed and mapped far below the diffraction limit with sub-attomole sensitivity. Its sensitivity and resolutions could be further enhanced not only by alleviating its inherent background scatterings and system noises but also by precision-engineering probe tips. Furthermore, more powerful THz pulse sources, if available, would also significantly improve THz s-SNOM sensitivity. We envisage a THz s-SNOM system with sub-attomole sensitivity and true nanoscale resolutions in the near future, pushing forward the frontier of THz technology by realizing the high-speed and ultrasensitive molecular-scale THz sensing and imaging of previously unattainable biological and biochemical materials and quantum matters as well as trace isomer and polymorphic impurities in organic crystals. This would manifest intriguing prospects of THz technology in many fields of research including biology, life science, chemistry, physics, materials science and engineering among many others.

## Methods

### Experimental setup

Our THz s-SNOM consisted of a homemade tapping-mode AFM system and a conventional THz-TDS system as described elsewhere^7,8^, with some modifications (see Fig. S1 in Supplementary Information). Briefly, an InAs wafer was used for generating the THz pulses. The AFM probe was located at the center of the sample where the THz pulse was focused by off-axis parabolic mirrors. The THz pulse was incident on the sample with an angle of 60° and was p-polarized. The scattered pulse was detected by a photo-conductive antenna (PCA) fabricated on a low-temperature-grown GaAs wafer. The output of the PCA was demodulated at the tapping frequency of the probe by a lock-in amplifier.

The AFM probe was fabricated from a 50-μm-diameter tungsten wire by electrochemical etching method. The effective diameter of the probe apex was approximately 600 nm to improve scattering efficiencies on samples with low refractive indexes (*e*.*g*., lactose). The probe was glued to a quartz tuning fork to perform tapping-mode AFM operation. To perform surface mapping, the sample was raster-scanned. The experimental setup was enclosed in a dry-air purged chamber. During the measurements, the dew point was maintained to be at less than −55 °C.

### Sample preparation

Crystalline lactose sample was purchased from Sigma-Aldrich, Inc. (Milwaukee, WI, USA). α-Lactose monohydrate (Product number: L8783) in the mixture of α-lactose (≥95%, w/w) and β-lactose (≤4%, w/w) anomers was selected for this study. HDPE sample was purchased from Micro Powders Inc. (Tarrytown, NY, USA; Product number: MPP-645XF). The crystalline lactose and HPDE in powder forms were mixed at an equal mass ratio with a pharmaceutical spatula without additional treatments for their homogeneity. The powder mixture was then pressed into a pellet under ambient conditions with an average thickness of ~3 mm for the THz s-SNOM measurements. Also, a pellet only with the crystalline lactose was prepared as a reference for conventional THz-TDS.

### Computed complex permittivity

Line dipole image method (LDIM)^8,26^ was used to compute the complex permittivity of lactose. Briefly, in LDIM, we assume the metallic probe is replaced by a sphere and its diameter corresponds to the effective diameter of the probe apex. Since the probe diameter (~600 nm) is much smaller than the THz wavelength, a quasi-electrostatic image theory is adopted to calculate the induced dipole moment distribution in the probe and substrate. From the integrated sum of the dipole moment distribution, the far-field amplitude and phase of the scattered field as well as *N*(*ω*) are calculated. To compute the local complex permittivity, a self-consistent nonlinear iteration was performed with theoretical *N*(*ω*) values for a given set of substrate permittivity. See Supplementary Information for details of the LDIM.

## Supplementary information

Supplementary Information
