# Supplementary material for: Computed terahertz near-field mapping of molecular resonances of lactose stereo-isomer impurities with sub-attomole sensitivity
Source: Sci Rep. 2019 Nov 15;9:16915. doi: 10.1038/s41598-019-53366-0 (PMC6858443; doi:10.1038/s41598-019-53366-0)
Supplement: Supplementary file 1 — Supplementary Information [file 41598_2019_53366_MOESM1_ESM.pdf]

Supporting information

Computed terahertz near-field mapping of molecular resonances of lactose stereo-isomer impurities with sub-attomole sensitivity

Kiwon Moon[1†], Youngwoong Do[1‡], Hongkyu Park[1§], Jeonghoi Kim[1§], Hyuna Kang[1¶], Gyuseok Lee[1], Jin-Ha Lim[1], Jin-Woo Kim[1,2] and Haewook Han[1*]

[1]Department of Electrical Engineering, Pohang University of Science and Technology, Pohang 37673, Republic of Korea
[2]Department of Biological and Agricultural Engineering and Institute for Nanoscience and Engineering, University of Arkansas, Fayetteville, Arkansas 72701, USA
[†]Present address: THz Basic Research, Electronics and Telecommunications Research Institute, Daejeon 34129, Republic of Korea
[‡]Present address: SK Hynix Inc., Icheon 17336, Republic of Korea
[§]Present address: Samsung Electronics, Suwon 16677, Republic of Korea
[¶]Present address: Korea Brain Research Institute, Daegu 41062, Republic of Korea
*emails: hhan@postech.ac.kr (H.H.)

1. **Experimental Set-Up of THz Scattering-Type Scanning Near-Field Optical Microscopy (THz s-SNOM)**

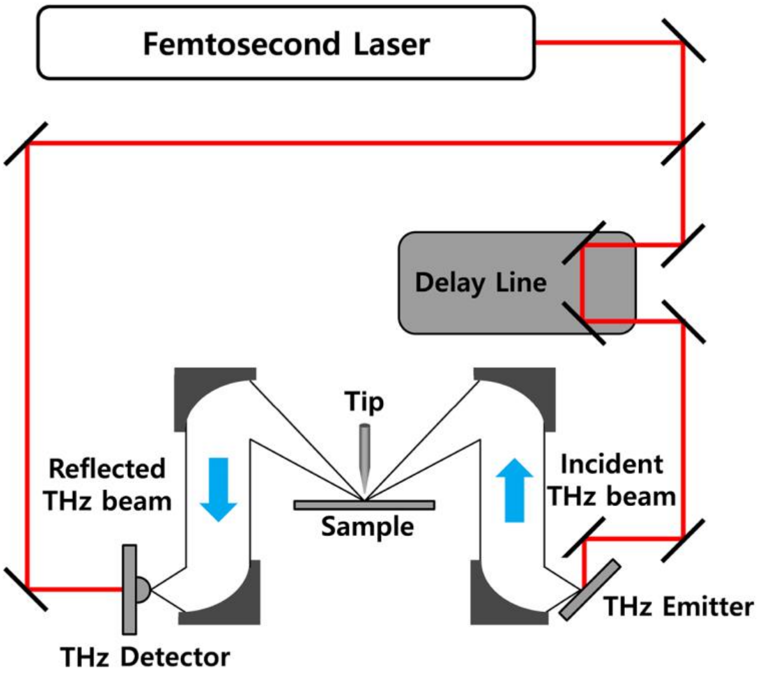

Figure S1. Schematic of experimental set-up of THz scattering-type scanning near-field optical microscopy (THz s-SNOM).

2. **Line Dipole Image Method (LDIM) to Compute Complex Local Permittivity of Crystalline Lactose Stereo-Isomers**

In the THz scattering-type scanning near-field optical microscopy (THz s-SNOM) in this study, the incident THz pulse is localized through a near-field probe that consists of a cylindrical probe shaft, taper zone, and sub-micrometer size apex at the end of the taper zone. The focused THz pulse is coupled to the probe and spatially localized by the taper zone. And the THz pulse is scattered into the free space after interacting with the sample under the apex within the near-field region defined by the size of the probe apex. In the scattering process, the probe shaft and tapered region simultaneously act as receiving and transmitting antennae that significantly influences the temporal and spectral shapes of the scattered THz pulse[1,2]. To fully understand the scattering process, it is necessary to understand the antenna effect and the near-field interaction at the probe apex. Although there have been studies on the antenna effect[1,2] and the near-field interaction between the probe and the substrates[3,4], no complete theoretical model is currently available.

Using the relative contrast $N(\omega)$, the extraction of complex permittivity is possible without considering the antenna effect because of the common factors, including the antenna response, are cancelled out in $N(\omega)$. The relative contrast can be fully determined by the near-field interaction at the end of the probe[3].

In the LDIM, the probe is modeled as a metal sphere on a semi-infinite planar substrate[3,4], as shown in Figure S2. Although the samples are inhomogeneous in many of practical applications, it is reasonable to assume a homogeneous semi-infinite material because the near-field interaction is strongly localized by the probe apex. In addition, the electro-static image theory is adopted in the LDIM, which is justified by the fact that the sphere diameter (~600 nm in this study) is much smaller than the wavelength of the incident THz wave.

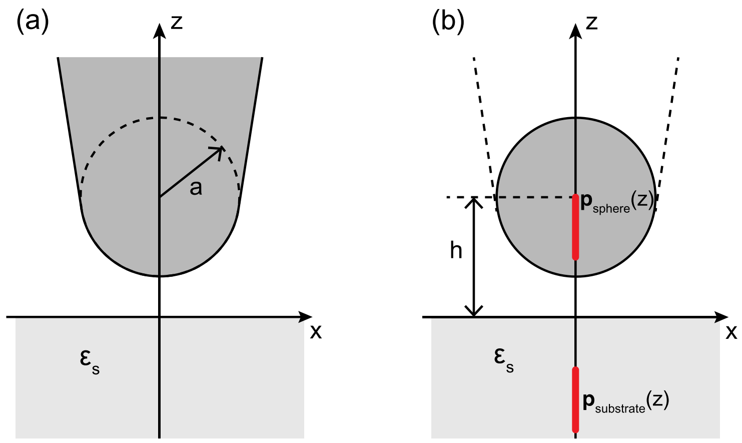

Figure S2. **THz s-SNOM probe-sample interactions. a**, Schematic of probe-sample system. The effective radius of the probe apex is *a* and the substrate index is ε_S. **b**, Probe approximated as a sphere with center height h. The height is dithered along the *z*-axis at frequency Ω.

The LDIM calculates the induced line dipole moment distributions in the metal sphere and the substrate, which are denoted by $\mathbf{p}_{sphere}(z,\varepsilon_S,h)$ and $\mathbf{p}_{substrate}(z,\varepsilon_S,h)$, respectively. As depicted in Figure S2, *z*, *h*, and ε_S denote the *z*-coordinate, height of the sphere center, and complex permittivity of the substrate, respectively. The calculated line dipole distribution satisfies electrostatic boundary conditions on the surfaces of the sphere and the substrate[3]. Near-field distribution in the sample can be calculated from the dipole distribution in the sphere, seen by the space below the sample surface. In the far-field region, the scattered field is calculated by the total dipole moment distribution that is obtained by integrating the dipole distributions in the sphere and the substrate. The scattered field is described by[5]:

$$\mathbf{S}(\mathbf{r},\varepsilon_S,h)=\frac{k^2}{4\pi\varepsilon_0}\mathbf{n}\times\left[\int_{-h}^{h}\left\{\mathbf{p}_{sphere}(z',\varepsilon_S,h)+\mathbf{p}_{substrate}(z',\varepsilon_S,h)\right\}dz'\right]\times\mathbf{n}$$

where **r** is the position vector of the observation point and **n** = **r**/*r*. Since the scattered field intensity in the far-field is proportional to the total dipole moment, far-field calculation is not required for the calculation of the relative contrast.

By the tip dithering and subsequent lock-in detection, we obtained the total dipole moment for time-varying $h(t)=a+g(1+\sin\Omega t)/2$, where *a*, *g*, and Ω denote the sphere radius, probe oscillation amplitude, and probe oscillation frequency, respectively. Because of the strong non-linear interaction between the probe and the substrate, the scattered field intensity is given by the multiple harmonic components:

$$|\mathbf{S}(\mathbf{r},\varepsilon_S,h)|=\sum_{m=1}^{\infty}S_m(\varepsilon_S)\cos(m\Omega t)$$

We used the first harmonic component ($S_1(\varepsilon_S)$) for computed mapping of the measured scattering signal because it can provide the highest signal-to-noise ratio with $S_1(\varepsilon_S)$ in our THz-NFM system. Based on the LDIM, $S_1(\varepsilon_S)$ can be numerically calculated, and $N(\omega)$ and $S_1(\varepsilon_A)/S_1(\varepsilon_B)$ can also be obtained by using local complex permittivity ε_A and ε_B, measured by our THz-NFM system. To extract the complex permittivity from the experimentally obtained $N(\omega)$, we derived inverse transfer functions to extract the real and imaginary parts of the permittivity from the experimental $|N(\omega)|$ and $\varphi(N(\omega))$. For this purpose, we first calculated theoretical $|N(\omega)|$ and $\varphi(N(\omega))$ values for a given range of $\varepsilon_A = \varepsilon_r + i\varepsilon_i$ using $\varepsilon_B = 2.353$ (the permittivity of HDPE), to make a look-up table shown in Table S1. The diameter of the sphere was set to be 600 nm, which is a good approximation of the actual effective radius of the probe apex.

Table S1. Calculated $|N(\omega)|$ and $\varphi(N(\omega))$ for a range of ε_A values defined by $\varepsilon_A = \varepsilon_r + i\varepsilon_i$

ε_r	ε_i	$|N(\omega)|$	$\varphi(N(\omega))$	ε_r	ε_i	$|N(\omega)|$	$\varphi(N(\omega))$
1.2	0	0.13290	0.00146	2.8	0	1.33397	0.00146
1.2	0.24	0.24977	1.02031	2.8	0.56	1.41947	0.31504
1.2	0.48	0.44359	1.30000	2.8	1.12	1.65504	0.56099
1.2	0.72	0.66379	1.42498	2.8	1.68	1.99596	0.72288
1.2	0.96	0.90896	1.49030	2.8	2.24	2.39730	0.81705
1.2	1.2	1.17522	1.52028	2.8	2.8	2.82876	0.86503
1.6	0	0.41892	0.00146	3.2	0	1.62278	0.00146
1.6	0.32	0.50520	0.59928	3.2	0.64	1.71083	0.27991
1.6	0.64	0.71190	0.94938	3.2	1.28	1.95484	0.50274
1.6	0.96	0.98054	1.13136	3.2	1.92	2.30949	0.65287
1.6	1.28	1.28723	1.22553	3.2	2.56	2.72845	0.74185
1.6	1.6	1.61877	1.26965	3.2	3.2	3.17378	0.78761
2	0	0.72458	0.00146	3.6	0	1.89810	0.00146
2	0.4	0.80657	0.44530	3.6	0.72	1.98850	0.25364
2	0.8	1.02221	0.75800	3.6	1.44	2.23970	0.45797
2	1.2	1.32014	0.94255	3.6	2.16	2.60528	0.59779
2	1.6	1.67198	1.04377	3.6	2.88	3.03566	0.68160
2	2	2.04938	1.09273	3.6	3.6	3.49040	0.72512
2.4	0	1.03292	0.00146	4	0	2.15782	0.00146
2.4	0.48	1.11602	0.36527	4	0.8	2.25019	0.23300
2.4	0.96	1.34187	0.64069	4	1.6	2.50701	0.42215
2.4	1.44	1.66490	0.81510	4	2.4	2.88057	0.55294
2.4	1.92	2.04551	0.91404	4	3.2	3.31881	0.63199
2.4	2.4	2.45442	0.96340	4	4	3.77978	0.67328

Note: that ε_B was assumed to be 2.353, the real permittivity of HDPE.

A multi-variable regression method was used to formulate a transfer function that extracts the complex permittivity of the substrate from a given pair of $|N(\omega)|$ and $\varphi(N(\omega))$. The mean-square errors of the obtained regression function were determined to be 5.7293×10^{-5} and 1.1859×10^{-5} for the real and imaginary parts, respectively. The extracted transfer function was applied to extract the complex permittivity from the measurements. In practice, we obtained the functions to calculate $\sqrt{\varepsilon_r^2+\varepsilon_i^2}$ and $\varepsilon_i / \varepsilon_r$ from $|N(\omega)|$ and $\varphi(N(\omega))$ for the best fit. The terms and coefficients of the regression are shown in Table S2, where $x = |N|$ and $y = \varphi$. The contour plots of the inverse functions (transfer functions) are shown in Figure S3.

Table S2. Regression terms and coefficients

	$\sqrt{\varepsilon_r^2+\varepsilon_i^2}$	$\varepsilon_i / \varepsilon_r$
const.	1.17121020	0.349042131
x	0.51952563	0.443373782
y	0.65963347	0.382444226
xy	-0.54223796	0.392143974
x^2	-0.06158182	-0.007220926
y^2	0.83263329	0.259093993
x^2y	-0.05983339	0.005512876
xy^2	-0.23529379	0.088715566
x^3	0.00296535	0.001509645
y^3	0.25795215	0.109697621

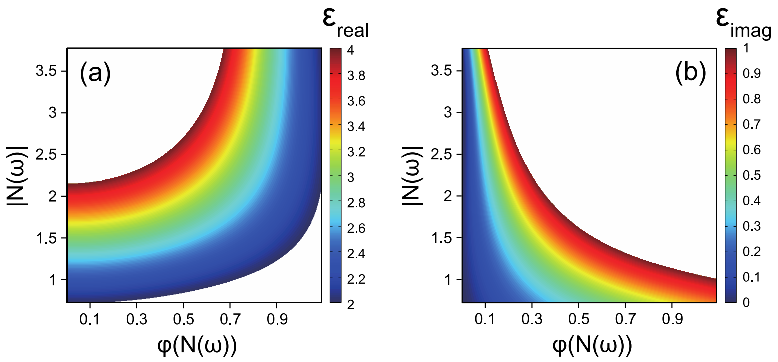

Figure S3. Contour plots of inverse functions. a,**b**, Real (**a**) and imaginary (**b**) parts of permittivity with $N(\omega)$.

3. Lorentz Fit of Complex Permittivity

The complex permittivity from s-SNOM measurement can be fitted to the triple Lorentz model, which is defined by the following equation:

$$\varepsilon(\omega) = \varepsilon_\infty + \sum_{n=1}^{3} \frac{\omega_{p,n}^2}{\omega_{0,n}^2 - \omega^2 - i\gamma_n \omega}$$

where $\omega_{0,n}$ and γ_n are the resonance frequency and the damping parameter, respectively.

For comparison, the Lorentz parameters for the reference TDS measurements are also shown. Three resonance peaks and damping parameters are summarized in Table S3. The peaks and damping parameters from s-SNOM and THz-TDS are reasonably in agreement. The values also agree with those obtained from previous measurements by a continuous-wave THz spectroscopy system[6].

Table S3. Extracted Lorentz parameters of crystalline-lactose stereo-isomers from TDS and THz s-SNOM measurements

Resonance and damping frequencies (THz)		n = 1 (α-Lactose)	n = 2 (β-Lactose)	n = 3 (α-Lactose)
$\omega_{0,n}$	TDS	0.531	1.196	1.371
	s-SNOM	0.530	1.188	1.369
$\gamma_{0,n}$	TDS	25.54	47.17	52.13
	s-SNOM	29.18	27.17	71.00

4. THz s-SNOM Detection Sensitivity

The detection sensitivity of THz s-SNOM was estimated under the assumptions that the probe-sample interaction volume is same as the THz near-field penetration volume and that lactose powder is fully saturated in the volume.

Estimation of the probe-sample interaction volume. The probe-sample interaction volume of our THz s-SNOM was estimated under the assumption that the probe-sample interaction volume is same as the THz near-field penetration volume, which was assessed through image analyses (Fig. S4). Specifically, we calculated the penetration depth of THz near-field at each point using the self-consistent LDIM to estimate the interaction volume of 8.066×10^{-4} μm^3 (= 8.066×10^{-16} cm^3) under the $1/e$ cutoff criterion.

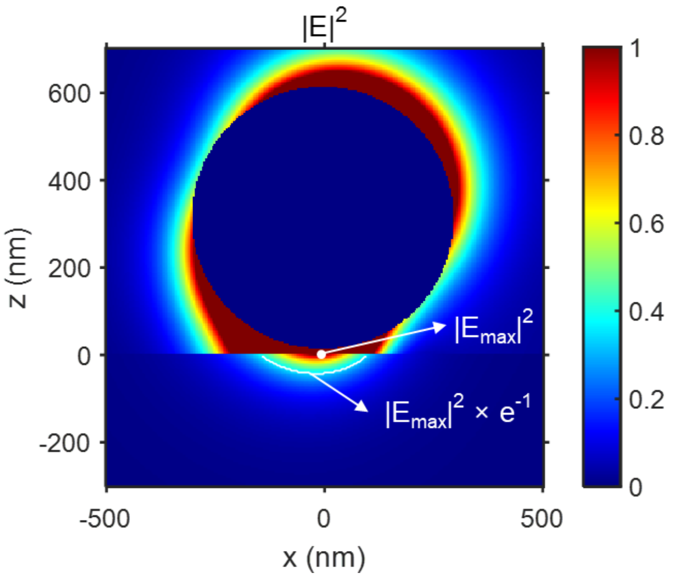

Figure S4. THz near-field power density distribution. The near-field was calculated by the line dipole image method (LDIM) using the quasi-electrostatic boundary condition. The interaction volume of 8.0659×10^{-4} μm^3 is enclosed by z = 0 and the white contour for $|E_{max}|^2 \times e^{-2}$ where E_{max} is the maximum electric field.

Estimation of Detection sensitivity of lactose anomers. Crystalline lactose sample purchased from Sigma-Aldrich, Inc. (Product number: L8783; Milwaukee, WI, USA) was in the mixture of α-lactose monohydrate (≥95%, w/w) and β-lactose (≤4%, w/w) anomers. Molecular weights and specific weights (*i.e.*, true densities) are 360.31 g/mole and 1.547 g/cm^3 for α-lactose monohydrate, respectively, and 342.30 g/mole and 1.590 g/cm^3 for β-lactose, respectively[7]. It was assumed that lactose powder is fully saturated in the probe-sample interaction volume of 8.066×10^{-4} μm^3 (= 8.066×10^{-16} cm^3) and that the volume of the trace impurities (≤1%, w/w) in the lactose sample is negligible. Under the premises, the amount of α-lactose in the interaction volume was estimated to be 1.199×10^{-15} g (= 3.327×10^{-18} mole) and that of β-lactose 5.047×10^{-17} g (= 1.475×10^{-19} mole). Hence, the detection sensitivities of our THz s-SNOM were 3.328 attomole of α-lactose and 0.147 attomole of β-lactose.

References

1. Wang, K. & Mittleman, D. M. Antenna effects in terahertz apertureless near-field optical microscopy. *Appl. Phys. Lett.* **85,** 2715-2717 (2004).
2. Chen, H. -T., Kraatz, S., Cho, G. C. & Kersting, R. Identification of a resonant imaging process in apertureless near-field microscopy. *Phys. Rev. Lett.* **93,** 267401 (2004).
3. Moon, K., Jung, E., Lim, M. Do, Y. & Han, H. Quantitative analysis and measurements of near-field interactions in terahertz microscopes. *Opt. Express* **19,** 11539-11544 (2011).
4. Moon, K. *et al.* Quantitative coherent scattering spectra in apertureless terahertz pulse near-field microscopes. *Appl. Phys. Lett.* **101**, 011109 (2012).
5. Jackson, J.D. Classical Electrodynamics (John Wiley & Sons, Inc, 1999).
6. Roggenbuck, A. et al. Coherent broadband continuous-wave terahertz spectroscopy on solid-state samples. *New J. Phys.* **12,** 043017 (2010).
7. Haynes, W. M., Lide, D. R. & Bruno, T. J. *CRC Handbook of Chemistry and Physics*: *A Ready Reference Book of Chemical and Physical Data*, 97th Ed. (CRC Press, 2016).
